# Inpatient Multidisciplinary Rehabilitation in Adult Spinal Cord Injury Without Neuroimaging Abnormality (SCIWNA): A Case Report

**DOI:** 10.7759/cureus.52077

**Published:** 2024-01-11

**Authors:** Igor Santos Neto, Tiago Ribeiro, Miguel Guimaraes, Margarida Rodrigues, Fatima Gandarez, Maria Cunha

**Affiliations:** 1 Physical Medicine and Rehabilitation, Centro de Reabilitação do Norte - Centro Hospitalar de Vila Nova de Gaia Espinho, Porto, PRT; 2 Spinal Cord Injuries, Centro de Reabilitação do Norte - Centro Hospitalar de Vila Nova de Gaia Espinho, Porto, PRT

**Keywords:** spinal cord injury without neuroimaging abnormality (sciwna), magnetic resonance imaging, neuroradiology, true sciwora, spinal cord injury

## Abstract

Spinal cord injury without radiographic abnormality is a condition primarily observed in the pediatric population. However, its occurrence in adults remains underreported. This case report aims to describe a rare instance of adult spinal cord injury without neuroimaging abnormality (SCIWNA) following a road accident in a 52-year-old woman, emphasizing the clinical nuances and management challenges associated with this condition.

The patient presented with tetraplegia (American Spinal Injury Association Impairment Scale D) with a neurological injury level at C4, exhibiting subtle improvements during inpatient care. Comprehensive examinations revealed conflicting clinical and imaging findings, leading to the diagnosis of SCIWNA. A tailored rehabilitation program involving a multidisciplinary team resulted in notable improvements in neuromotor function, gait, and activities of daily living.

The mechanisms behind SCIWNA in adults remain debated, possibly involving pre-existing spinal pathologies exacerbated by trauma. Neurological deficits can range from minor sensory issues to severe tetraplegia with unpredictable times of onset. Diagnostic challenges persist due to normal imaging results despite clinical symptoms. Treatment guidelines lack consensus, encompassing conservative approaches, steroid administration, and surgical interventions in select cases.

This rare case of SCIWNA underscores the diagnostic complexity when clinical spinal cord injury contrasts with normal neuroimaging. This report highlights the significance of clinical assessment and the evolving landscape in diagnosing SCIWNA in adults. In addition, the absence of a standardized management protocol emphasizes the need for individualized strategies tailored to patient-specific needs, warranting further research and consensus-building among healthcare professionals.

## Introduction

Spinal cord injury without radiographic abnormality (SCIWORA) is a syndrome that occurs when the spinal cord sustains neural damage during a traumatic event without positive radiographic findings [1]. It was first described in 1974 when Burke presented a series of 29 children treated at Rancho Los Amigos Hospital with traumatic paraplegia under the age of 13 years [2]. Since then, new and improved imaging methods have been developed.

According to different studies, the incidence of SCIWORA is highly variable. It has been more commonly reported in children under the age of one year [3] than in adults (6% to 32% vs. 9% to 14%, respectively) and typically involves the cervical cord [4].

The majority of SCIWORA cases involve spinal cord injury in which both X-ray and CT scans demonstrate no imaging changes. However, in these cases, an MRI scan can identify injuries such as spinal cord edema. Therefore, a new concept arises, i.e., spinal cord injury without neuroimaging abnormality (SCIWNA), where no imaging changes are documented in traditional imaging methods (X-ray and CT scan) or on MRI [5].

This case report describes a clinical case of an adult patient diagnosed with SCIWNA. We aim to bring awareness to the management of this rare and unique condition, demonstrating the necessity of a well-performed physical evaluation and rehabilitation program to improve the patient’s well-being.

## Case presentation

A 52-year-old woman with no significant medical or surgical history and autonomously performing activities of daily living (ADLs) suffered a road accident that resulted in a spinal cord injury (SCI). Neurological levels and severity of impairment were graded according to the American Spinal Injury Association International Standards for Neurological Classification of Spinal Cord Injury (ASIA/ISNCSCI) (Appendices 1 and 2).

Upon arrival at the hospital, the initial evaluation established a diagnosis of tetraplegia American Spinal Injury Association Impairment Scale D (AIS D) with a neurological injury at C4. On physical examination, the patient had no language domain deficits, joint range of motion limitations, or visible muscular mass atrophies.

However, osteotendinous reflexes were hyperreactive, with a large reflexogenic area present in the right upper limb and lower limbs, and the Babinsky reflex was indifferent bilaterally. Regarding muscular strength assessment, according to the Medical Research Council (MRC) score, the myotomes of the upper limbs (C5-C7) were graded 4, C8 graded 3 on the right and 4 on the left, and T1 graded 4 bilaterally. No changes were observed on the lower limbs (L2-S1) except at L2 on the right, graded as 4 out of 5.

The patient presented with hypoesthesia at C5 bilaterally and anesthesia at C8 on the right. Additionally, significant dynamic balance and gait impairments were noted. For mobility, the patient used a wheelchair that she could self-propel with bilateral support.

There were no other significant clinical changes. Several imaging studies were performed. MRI of the cervical spine showed no spinal cord sign changes. Additionally, fracture or bone contusion, disc hernias, and spinal cord compression were not observed. A CT scan of the brain described no acute traumatic injuries, and the cervico-dorso-lumbar spine CT scan was unremarkable. The spinal canal had normal amplitude, and no recent traumatic injuries, namely, fractures, joint dislocations/subluxations, perivertebral soft tissue thickenings, or intracanal blood collections, were found. At C6-C7, discrete hypertrophy of posterior joints on the right was registered. A posterior disc-osteophyte complex at T1-T2 attenuated the anterior subarachnoid space without contact with the spinal cord and endocanal space conflict. There were no other changes. Neuromotor and functional evolution during acute inpatient care were subtle, with a slight improvement in the neuromotor condition (improvement in the right hemibody at C8 and recovery of sensitivity below the sensory level of C6), and no changes to any other domain were described. Afterward, the patient came to our rehabilitation center and enrolled in an intensive rehabilitation program to improve neuromotor and functional outcomes.

At admission, the multidisciplinary team performed a comprehensive functional and clinical examination. The patient complained of bilateral paraesthesia on the fingertips without other significant complaints. On physical examination, the patient had no language domain deficits, joint range of motion limitations, or visible muscular mass atrophies. Hoffman’s reflex test was positive bilaterally, osteotendinous reflexes were hyperreactive with a large reflexogenic area, and clonus was present and easily exhaustible in the ankle joint. Babinsky reflex was positive only on the left side. Myotomes were graded 4 out of 5 during muscular strength assessment of the upper limbs (C5-T1). No changes were documented on the lower limbs (L2-S1). Thermo-algic on the right was normal up to the C4 level, with hypoesthesia at C5-T1 and preserved below those levels; on the left, hypoesthesia was documented at C5 only. Light touch, on the right, was preserved up to C4, with hypoesthesia at C5-T1; on the left, there was no change up to C4, with hypoesthesia at C5 and normal below.

Additionally, the patient had significant dynamic balance and gait impairments. Regarding mobility, the patient was in a wheelchair, which she could propel herself, with no other clinical changes. Our examination confirmed a diagnosis of tetraplegia AIS D with a neurological injury at the C4 level. Given the absence of a correlation between clinical and radiological findings, we assumed the diagnosis of adult SCIWNA.

A patient-tailored multimodal rehabilitation program was designed to enhance neuromotor and functional outcomes with gait recovery using an assistive device, ADL independence improvement, neuropathic pain control, manual dexterity improvement; psychosocial assessment; and assistive devices assessment, prescription, evaluation, and management of the ability to drive with safety.

To achieve these goals, a multidisciplinary team comprising a physiatrist, a physiotherapist, an occupational and speech therapist, a rehabilitation nurse, and a neuropsychologist was needed.

After weeks of an intensive multimodal rehabilitation program, we obtained favorable outcomes in gait optimization without needing assistive devices and with minimal claudication. Additionally, following balance and gait training, we achieved optimal levels of static and dynamic balance, allowing the patient to be independent on transfers and assume the orthostatic position quickly. The patient was able to tolerate maximal efforts measured on the Borg scale.

We also achieved maximum independence on ADL at discharge, with a relevant improvement of the Functional Independent Measure (FIM) score and other scales, such as strength assessment and thermo-algic (Tables 1-4).

**Table 1:** Findings of the objective scales. FIM = Functional Independent Measure; WISCI II = Walking Index for Spinal Cord Injury; ARAT = Action Research Arm Test; R/L = right/left; 9-HPT = 9-Hole Peg Test

Objective scales	At admission	At discharge
FIM score	82/126	125/126
WISCI II	20/20	20/20
ARAT (R/L)	40/57–46/57	57/57–57/57
9-HPT (R/L)	25/22 seconds	18/21 seconds

**Table 2:** Evolution of muscle strength from admission to discharge using the MRC scale. MRC = Medical Research Council; graded as 5 (normal), 4 (movement against gravity and resistance), 3 (movement against gravity over the entire range), 2 (movement of the limb but not against gravity), 1 (visible contraction without movement of the limb), and 0 (no visible contraction).

Strength assessment (MRC)	At admission	At discharge
C5–C8	4/5	5/5
C8–T1	4/5	4+/5
L2	5/5	4/5
L3–S1	5/5	5/5

**Table 3:** Evolution of thermo-algic sensitivity from admission to discharge using the ISNCSCI scale of ASIA. R/L = right/left sensory grading: 0 = absent, 1 = altered, either decreased/impaired sensation or hypersensitivity, 2 = normal; ISNCSCI = International Standards for Neurological Classification of Spinal Cord Injury; ASIA = American Spinal Injury Association International

Thermo-algic (R/L)	At admission	At discharge
C4	2/2–2/2	2/2–2/2
C5	1/2–1/2	2/2–1/2
C6–T1	1/2–2/2	1/2–2/2
T2–S1	2/2–2/2	2/2–2/2

**Table 4:** Evolution of light touch sensitivity from admission to discharge using the ISNCSCI scale of ASIA. R/L = right/ left sensory grading: 0 = absent, 1 = altered, either decreased/impaired sensation or hypersensitivity, 2 = normal; ISNCSCI = International Standards for Neurological Classification of Spinal Cord Injury; ASIA = American Spinal Injury Association International

Light touch (R/L)	At admission	At discharge
C4	2/2–2/2	2/2–2/2
C5	1/2–1/2	1/2–1/2
C6–T1	1/2–2/2	1/2–2/2
T1–S1	2/2–2/2	2/2–2/2

No neurologic changes were reported. However, adjustments regarding bowel movements were made to ensure regular transit while undergoing the rehabilitation program. Recommendations were provided for continuous outpatient rehabilitation.

The patient was followed up at our rehabilitation center, maintaining all the capabilities acquired during the intensive inpatient rehabilitation program. She mentioned only increased frequency of limb member spasms, which merited follow-up assessment in a specialized consultation. She underwent physiotherapy follow-up at an outpatient clinic.

## Discussion

SCIWORA is well recognized in the pediatric population. However, it is a rare phenomenon in adults, with fewer than 100 cases reported, according to Sharma et al. [6]. It is more common in males (68.5%), with a peak in reproductive ages. Despite being considered a rare condition, the prevalence is increasing steadily due to improved survival rates in the acute and chronic stages of the injury. Depending on the patient’s injuries’ age, level, and severity, life expectancy ranges from four to more than 50 years after injury [7].

The mechanism of SCIWORA in adults is controversial. However, the development of degenerative pathologies before the trauma, including cervical spinal stenosis, disc herniation, ossification of the posterior longitudinal ligament, and calcification of ligamentum flavum, may be closely related to the mechanism of the lesion. Likewise, patients with soft tissue injuries, such as anterior longitudinal ligament or ligamentum flavum injury, show less ability to improve. The external force reduces the storage capacity of the spinal canal, compressing the spinal cord, which is consistent with the MRI findings of patients after injury [8].

Furthermore, after Pang and Pollack described four mechanisms of SCIWORA, including flexion, hyperextension, longitudinal distension, and ischemia [9], other studies showed that ligamentous injuries, disc prolapses, intramedullary lesions, and hematomas are among the most common etiologic findings in adults [10].

Patients diagnosed with SCIWORA may experience a broad spectrum of ND [4]. Usually, neurological deficits (NDs) tend to be more severe in the upper extremities [11]. Moreover, the onset of clinical symptoms may be delayed from a few minutes to two days after injury in half of the patients. This latency is thought to exist because of micro traumas to the spinal cord from striking against the unstable vertebrae, causing edema or a developing hematoma around the spinal cord [12].

Prompt identification and intervention are imperative to enhance patient outcomes and mitigate the potential occurrence of severe consequences.

Moreover, as the physical examination is limited and symptom onset may be delayed, conventional X-rays should be performed at the first approach (lateral spine, anteroposterior, oblique, and open mouth). A CT scan is the most accurate method for detecting bone pathology, namely, injuries of the posterior arch or lateral mass of the vertebra, atlas, and odontoid process, which are usually poorly visible on standard X-rays. After excluding a spinal fracture, SCIWORA is suspected, and an MRI should be performed. This diagnostic modality is the gold standard for the assessment of SCIWORA owing to its unparalleled efficacy in directly evaluating the spinal cord, enabling the identification of underlying causes through the discrimination of extramedullary factors (such as disc herniation, canal stenosis, lesions of the anterior joint vertebral ligament, posterior ligament complex, and intracanal hematoma) and intramedullary factors (including edema, contusion, and hemorrhage) [13]. Further, MRI facilitates the detection of bone marrow edema within traumatized vertebrae, a feature not discernible on CT scans [14].

This diagnostic process should consider a broad differential diagnosis, including potential causes such as embolism resulting from vertebral artery occlusion linked to cardiovascular conditions such as endocarditis, cardiac arrhythmia, persistent foramen ovale, arteritis, or bleeding disorders. Additionally, the diagnostic evaluation should rule out the presence of acute or chronic myelitis.

Clinical guidelines for the treatment of SCIWORA should be established. Conservative strategies involve neck immobilization and the administration of steroids in high dosages [15]. Regardless of the MRI findings, the primary therapeutic approach for spinal injury is external immobilization of the spine, lasting up to 12 weeks, and avoidance of activities with an increased risk of injury for a minimum of six months [12].

When there is a likely diagnosis based on clinical findings and CT scan results, methylprednisolone may be administered before definitive radiological imaging by MRI [16]. The administration of a bolus dose of methylprednisolone (30 mg/kg) within three hours of injury, followed by a continuous infusion of methylprednisolone at a rate of 5.4 mg/kg per hour for 24 hours, or an alternative regimen involving a bolus dose within eight hours of injury followed by a 48-hour infusion, has been associated with significantly improved neurological outcomes [17].

Surgical intervention may be considered when extraneural abnormalities, such as ligamentous injury or cord compression, require operative intervention [18].

The rarity of SCIWORA in the adult population is a diagnostic challenge for clinicians who must rely on clinical assessment to identify spinal cord injuries in patients with NDs but normal X-ray films and CT scans. Furthermore, some patients diagnosed with this condition have normal findings on MRI, leading some authors to consider that these cases should be better classified as “true SCIWORA” or SCIWNA [5].

In these patients, in particular, diffusion-weighted imaging (DWI) can show hyperintensity with diffusion restriction for acute infarction, further supporting the diagnosis [19].

Since the systematic reviews of 2013 and 2015, SCIWORA has been increasingly recognized. This trend allows us to draw parallels with our case report. Similar to documented literature describing the cervical spine, particularly between C5 and C6, as the most frequently affected injury site, our patient also presented with a neurological injury level close to C4. Likewise, the neurological impairment scale score of AIS D in our patient aligns with previous records as the most common AIS grade.

On the other hand, based on the results of complementary diagnostic modalities and considering the absence of imaging alterations, our patient should be classified as having SCIWNA, a rare entity in adults and underreported in the literature. This topic has sparked discussions due to potential confusion in the use of diagnostic terms, given that this is an evolving field heavily reliant on advancements in MRI, particularly in its ability to identify details that contribute to the complexities and nuances of spinal cord injury diagnoses.

In this context, it might be relevant to consider adopting a classification system such as the Boese and Lechler classification system (further description in Table 5).

**Table 5:** Boese and Lechler classification.

Type	Boese and Lechler classification
Type 1	No detectable abnormalities
Type 2a	Extraneural abnormalities
Type 2b	Intraneural abnormalities
Type 2c	Extraneural and intraneural abnormalities

Rather than modifying the term SCIWORA, this system allows for a straightforward and unambiguous categorization of the type of spinal cord injury within the traditional SCIWORA framework.

## Conclusions

Adults often present degenerative changes to the spinal cord, resulting in predisposing spinal stenosis, such as instability of vertebral ligaments, preexisting cervical spondylosis, and herniation of a disk or hematoma around the spinal cord. None of these show on X-ray imaging.

We concluded that this is a rare case of SCIWNA. This article highlights proper clinical diagnosis when there is clinical evidence of spinal cord injury but no neuroimaging changes. No definitive treatment protocol has been established; a kaleidoscope of opinions regarding surgical and conservative management still exists among clinicians.

## Figures and Tables

**Figure 1:**
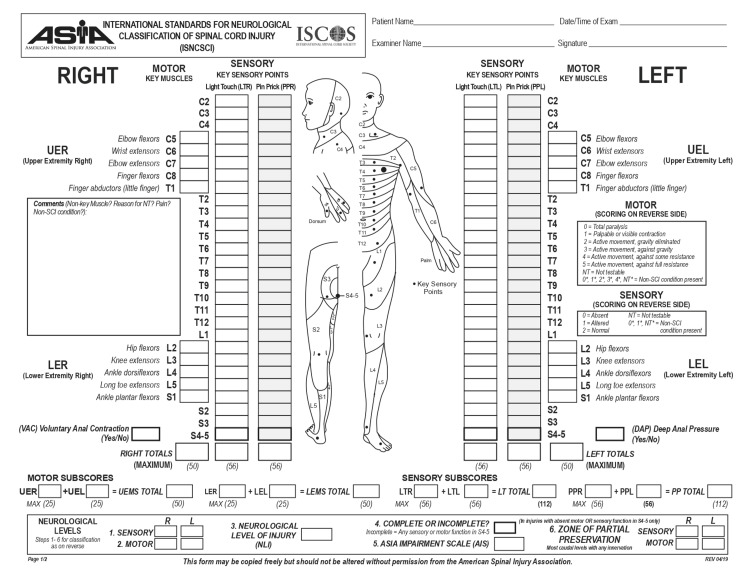
American Spinal Injury Association (ASIA) Injury Scale. Source: American Spinal Injury Association: International Standards for Neurological Classification of Spinal Cord Injury Front Page.

**Figure 2:**
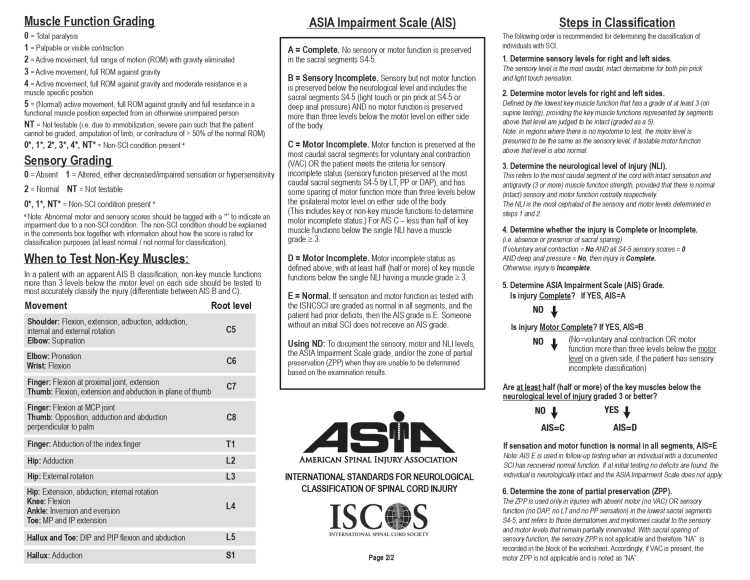
American Spinal Injury Association (ASIA) Injury Scale. Source: American Spinal Injury Association: International Standards for Neurological Classification of Spinal Cord Injury Back Page.
